# Objective measurement of oral function in adults with spinal muscular atrophy

**DOI:** 10.1186/s13023-023-02688-4

**Published:** 2023-05-03

**Authors:** T. Kruse, S. Shamai, D. Leflerovà, B. Wirth, R. Heller, N. Schloss, H. C. Lehmann, S. Brakemeier, T. Hagenacker, B. Braumann, G. Wunderlich

**Affiliations:** 1 Department of Orthodontics, Faculty of Medicine and University Hospital Cologne, University of Cologne, Kerpener Str. 32, 50931 Cologne, Germany; 2 Center for Rare Diseases Cologne, University of Cologne, Cologne, Germany; 3 Department of Prosthetic Dentistry, Faculty of Medicine and University Hospital Cologne, University of Cologne, Kerpener Str. 32, 50931 Cologne, Germany; 4 Institute of Human Genetics, University of Cologne, Kerpener Str. 34, 50931 Cologne, Germany; 5 Center for Molecular Medicine Cologne (CMMC), University of Cologne, Cologne, Germany; 6 Present Address: Genetic Health Service NZ–Northern Hub, Auckland District Health Board, Auckland City Hospital, 90-102 Grafton Rd, Grafton, NZ-Auckland, 1010 New Zealand; 7 Department of Neurology, Faculty of Medicine and University Hospital Cologne, University of Cologne, Kerpener Str. 62, 50937 Cologne, Germany; 8 Department of Neurology, Center for Translational Neuro- and Behavioral Sciences (C-TNBS), University Hospital Essen, Essen, Germany

**Keywords:** Spinal muscular atrophy, Bulbar neuromuscular function, Outcome measures, Bite force, Tongue pressure, Fatigability, Fatigue, Endurance, Validity

## Abstract

**Background:**

Impairment of bulbar function in adult individuals with spinal muscular atrophy (SMA) usually is not assessed by established motor scores. Measurements of oral function including quantitative muscle and endurance tests are able to detect subtle changes. The aim of this study was to systematically evaluate the measurement of maximum bite force and endurance, maximum tongue pressure and endurance, as well as maximum mouth opening in adult individuals with SMA types 2 and 3.

**Methods:**

Data from oral function tests in 43 individuals were analyzed. Differences in oral function between individuals with different SMA types and numbers of *SMN2* copies were tested. Spearman´s rho correlations among oral function measures themselves as well as with established clinical outcome scales were analyzed.

**Results:**

The absolute maximum measures of oral function (maximum bite force, maximum tongue pressure, maximum mouth opening) were able to discriminate between individuals with different SMA types, individuals with a different number of *SMN2* copies and with different walking abilities. The pairwise correlations of the absolute maximum measures of oral function were fair to moderate in size; the same was true for their correlations with the established motor scores. All correlations assessing endurance measures of oral function were weaker and statistically insignificant.

**Conclusions:**

Among the oral function tests maximum tongue pressure and maximum mouth opening are particulary promising as clinical and sensitive outcome measures for clinical trials. Oral function tests may supplement existing motor scores, in particular concerning specific questions about bulbar function or in severely affected non-ambulatory individuals where mild (treatment-related) changes would otherwise remain undetected.

*Trial registration* DRKS, DRKS00015842. Registered 30 July 2019, https://drks.de/search/de/trial/DRKS00015842

## Background

5q-associated spinal muscular atrophy (SMA) is a rare disease with an incidence of 1 in 7.000 live births in Germany, based on newborn screening [1, 2]. This hereditary autosomal recessive neuromuscular disorder is characterized by a progressive degeneration of motor neurons in the spinal cord. Biallelic deletions and/or point mutations of the survival of motor neuron 1 (*SMN1*) gene lead to a SMN protein deficiency [3, 4]. The consequences are muscular atrophy and weakness, as described in detail for axial and proximal muscle groups. While early bulbar symptoms have not yet been systematically recorded, bulbar muscles may be affected once the brainstem motor nuclei are involved [5–7]. The survival motor neuron 2 (*SMN2)* gene is a homologous copy of *SMN1.* Its number of copies inversely correlates with disease severity, and *SMN2* is the target of pharmacotherapeutic approaches [8–11]. Nusinersen, an antisense oligonucleotide drug for SMA therapy was the first causative treatment correcting the splicing of *SMN2* pre-mRNA and thus increasing the production of the SMN protein [12, 13].

The clinical spectrum of SMA is very broad. According to age of onset and severity, best achieved motor milestone and life span, the different subtypes 0–4 are classified [14]. Not just since therapy options have been available, there is an urgent need for sensitive clinical tools to observe relevant motor skills including bulbar function in order to precisely represent the functional status of individuals with SMA.

Progressive weakness and degeneration of bulbar muscles in SMA patients become evident in impaired muscle strength and increased fatigability in oral functions. Noticeably, for the patients, it affects their speaking, chewing, swallowing and maximum mouth opening [7, 15, 16]. Clinically, it is observed that oral functions are usually later and less severely affected than axial and proximal muscles in patients with SMA type 1 and 2 [16]. Electrophysiological and video fluoroscopic examinations, questionnaires, and MRI scans confirmed reduced oral functions in these patients [7, 17]. Approaches to quantitatively assess oral function in untreated SMA patients showed that maximum bite force was reduced by 19—50% compared to healthy controls [7, 15]. Reported values of maximum bite force in healthy subjects greatly vary, due to physiological and methodological factors [18], with a landmark of 560 N for the method used in this study (bite force endurance: 65 s; own data on healthy subjects, not reported here). Tongue pressure has already been suggested to be associated with bulbar and upper limb function in SMA and has been presented as a useful biomarker with mean values of 15.3 ± 6.4 kPa in SMA patients versus 37.3 ± 9.6 kPa in healty controls [19]. A physiological active mouth opening can be considered to be about 55 mm and has been shown to be clearly reduced to 38.9 ± 15.3 mm in SMA patients [7].

In patients with SMA, minor bulbar and oral symptoms may occur at earlier stages than currently assumed [19], which is one reason why they should be routinely recorded. There are other important reasons to routinely measure oral function in SMA patients. First, restrictions in oral function have a direct impact on the patient's quality of life [17, 20]. Second, due to a restricted mouth opening and reduced bite force / tongue pressure patients are at risk of serious adverse events, e.g. complications with intubation, severe choking due to impaired mastication or the risk of aspiration pneumonia [16, 21].

In severely affected adult individuals with residual motor function, measurements of oral function can provide valuable information on progressively impaired bulbar function. This may be particularly helpful when existing motor function scores reach their limits due to immobility of the patients. Representing only trunk and extremity function, the Hammersmith Functional Motor Scale (HFMSE) or the Revised Upper Limb Module (RULM), which are probably the most widely used motor scores in late-onset SMA patients, are at risk to miss out possible changes at the extreme ends of the spectrum of physical abilities [22–25]. Despite its potential, measures of oral function or more general bulbar function in SMA have not yet been validated or standardized. To capture impairment in oral function in different ways, measurement parameters should complement each other.

The Bogenhausener Dysphagia Score (BODS), the Sydney Swallow Questionnaire or the bullbar subscore of the Amyotrophic Lateral Sclerosis Functional Rating Scale Revised (ALSFRS-R) give first insights into parts of the bulbar function. However, these established scales depend on subjective evaluation, produce ordinal data with a limited response range, or are inadequate to cover the whole bulbar spectrum in SMA. A promising avenue to address these shortcomings is the quantitative muscle testing of oral function. The combined measurement of maximum bite force, maximum tongue pressure and maximum mouth opening covers a large part of bulbar muscle function [17]. The complementary measurements can detect small changes in bulbar function providing data over a continuous range. A pilot study confirmed the feasibility of maximum bite force measurements in two severely affected SMA patients during causative treatment, and provided first evidence of changes in bite force over the period of one year [26].

The discriminating power of combined oral function measures can be further increased by measuring muscle endurance. Endurance is defined as the prolonged maintenance of a constant or self-regulated force level [27, 28]. Even though considered less reliable than measures of maximum muscle strength [29], the measurement of oral function endurance is an important additional dimension of physical impairment in SMA. Muscle endurance is also targeted for therapeutic interventions [30–34]. Although the causes of fatigue in SMA patients have not yet been fully elucidated, and the associated disability hinders the patients’ daily life activities, endurance as an outcome measure has received limited attention [30–33]. Studying endurance in bite force and tongue pressure will help to identify factors associated with fatigability.

The aim of this study was to examine and systematically evaluate oral function tests including maximum bite force and endurance, maximum tongue pressure and endurance, as well as maximum mouth opening in adult SMA patients. We assessed the diagnostic potential of oral function tests (1) by evaluating their ability to differentiate between groups with different SMA-specific characteristics (SMA type, *SMN2* copy number, ambulatory status), (2) by identifying the extent to which the different measures of oral function are measuring the same construct, and (3) by examining the degree to which the newly introduced scores are consistent with established instruments measuring motor function.

## Material and methods

### Subjects

Initially, 44 SMA individuals were recruited into this study. According to the study protocol subjects were excluded if they showed significant respiratory compromise, a maximum mouth opening of less than 8 mm, or multiple missing teeth in the posterior region. The exclusion criteria applied to one out of the 44 individuals, who was excluded due to an extremely restricted mouth opening. A total of 43 adult individuals with genetically confirmed 5q-SMA were included for this study from the Departments of Neurology at the University Hospitals of Cologne and Essen, Germany. The sample size was not calculated, but determined by the number of eligible individuals willing to participate.

The study was approved by the Ethics Committees of the Medical Faculties of the two sites and conducted in accordance with the declaration of Helsinki (Reference Number Cologne: 19–1137; Reference Number Essen: 21–9851-BO). Each subject provided informed consent. Information on SMA type and *SMN2* copies were derived from the patients' medical records.

### Testing

Maximum bite force, bite force endurance, maximum tongue pressure, tongue pressure endurance, and maximum mouth opening were measured in accordance with the study protocol by Kruse and coworkers [35]. Oral function tests in each SMA patient were performed twice: two measurements were scheduled within one week, with a minimum of two days in between. No nusinersen application or other medical intervention was scheduled within these days. The tests were conducted prospectively at two sites by one of three dentists (DL, AC, TK) who had been previously trained in the method on healthy probands. For both study sites, standardized administration procedures and order of evaluation were set for each measure analyzed. Subjects were evaluated with the established motor scores as part of the routine evaluation during patients’ visits for nusinersen injection or as part of the natural history evaluation: HFMSE, RULM, ALSFRS-R and 6MWT (6-Minute-Walk-Test) were rated by physiotherapists, who had been trained in standardized therapy evaluation. BODS was carried out as part of the study by speech therapists, who were familiar with the evaluation of dysphagia in neuromuscular diseases.

To measure maximum bite force and bite force endurance, a piezoelectric sensor system consisting of a T-Scan sensor covering the entire dental arch and the I-Scan software (Tekscan, Inc., South Boston, MA) was used. Prior to the first measurement, the surface of the sensor was adjusted to the individual’s dental situation using dental silicon as described by Kruse and coworkers [35]. Testing the maximum bite force, individuals were asked to bite three times with maximum force for a duration of three to four seconds, with pauses of at least 30 s to avoid muscle fatigue. The highest score of maximum bite force (in Newton) was used for analysis. For the endurance test, individuals were asked to hold the adduction at 60% of the previously determined maximum bite force for as long as they could. The time (in seconds) until bite force dropped below 30% of the previously determined maximum bite force was used as an outcome value for further analysis.

Maximum tongue pressure and tongue pressure endurance were measured using a handheld device (IOPI Medical LLC, Carnation, WA: Iowa Oral Performance Instrument) with a single air-filled bulb tongue array, which was placed on the tongue blade in a predefined position: 10 mm posterior of the tongue tip and 10 mm anterior to the circumvallata papilla [36]. Individuals were advised to press their tongue against the air-filled bulb three times with maximum force for a duration of three to four seconds, with a 30 s pause between each repetition. The highest score of maximum tongue pressure (in kilopascal) was used for analysis. For the endurance test, individuals were asked to hold the muscle force at 60% of the previously determined maximum tongue pressure as long as they could. Again, time (in seconds) was recorded in which patients’ values dropped from 60% to 30% of their maximum value. Active mouth opening was measured at the mesioincisal angle of the upper and lower front teeth by a ruler registering the maximum distance (in millimeter) without reported pain.

### Inter- and intra-rater reliability

During additional measurements on healthy subjects inter- and intra-rater reliability was established using intraclass correlation coefficients (ICC). Inter-rater reliability was determined based on measurements by two raters (trained dentists DL and AC) alternately rating the same subjects within a one-week period (14 subjects overall for bite force and 7 subjects overall for tongue pressure). Intra-rater reliability was determined based on measurements by three raters (trained dentists DL, AC, and TK), each of whom rated the subjects twice within a given week (43 subjects overall for bite force and 33 subjects overall for tongue pressure).

### Statistical analysis

The outlined testing procedure resulted in patient-specific data on five outcomes from two visits. Distribution of the data was examined for normality. As all outcome variables failed to withstand the Kolmogorov Smirnoff or Shapiro–Wilk test, no normal distribution could be confirmed. For each outcome, the mean across the first and second measurement was used for analysis in order to reduce bias due to training effects or fluctuations depending on daily form [38, 39].

Discriminant power of oral function tests was examined via Wilcoxon rank-sum tests assessing distributional differences between different patient groups (SMA type 2 vs. 3, 3 vs. 4 *SMN2* copies, non-ambulatory vs. ambulatory). After alpha adjustment (Bonferroni correction), results were deemed statistically significant at a level of *p* < 0.017.

Correlations between the outcomes of the different oral function tests were assessed by means of Spearman´s rho (*ρ*). Results were deemed statistically significant at a level of *p* < 0.05.

Correlations among oral function tests and the clinical outcome scales BODS, HFMSE, RULM, ALSFRS-R and 6MWT were assessed by means of Spearman´s rho (*ρ*) and the treshold for statistical significance was set at *p* < 0.01 applying a Bonferroni correction for multiple testing. Since the 6MWT is only measured for ambulatory patients, non-ambulatory patients were defined as reaching a distance of 0 m for statistical analysis. The strength of all correlations was classified as none, poor, fair, moderate, very strong or perfect according to the definition introduced by Chan Y [40]. All statistical analyses were conducted using the statistical software SPSS 28.0.1.0 (IBM, SPSS statistics version 28.0.1.0, Chicago, IL, USA).

## Results

### Inter- and intra-rater reliability

Repeated measurements in healthy subjects indicated very good inter- and intra-rater reliability of the oral function measures, according to conventional guidances [37]. Intraclass correlation coefficients assessing interrater reliability were ‘almost perfect’ to ‘excellent’, ranging at 0.81 for maximum bite force, 0.95 for bite force endurance and 0.93 for maximum tongue pressure. For tongue pressure endurance the ICC of 0.67 showed ‘substantial agreement’. The intra-rater reliability of maximum bite force, bite force endurance, maximum tongue pressure and tongue pressure endurance were ‘excellent’: ICC values ranged at 0.94 for maximum bite force, 0.92 for bite force endurance, 0.95 for maximum tongue pressure and 0.96 for tongue pressure endurance.

### Main results

**Fig. 1:**
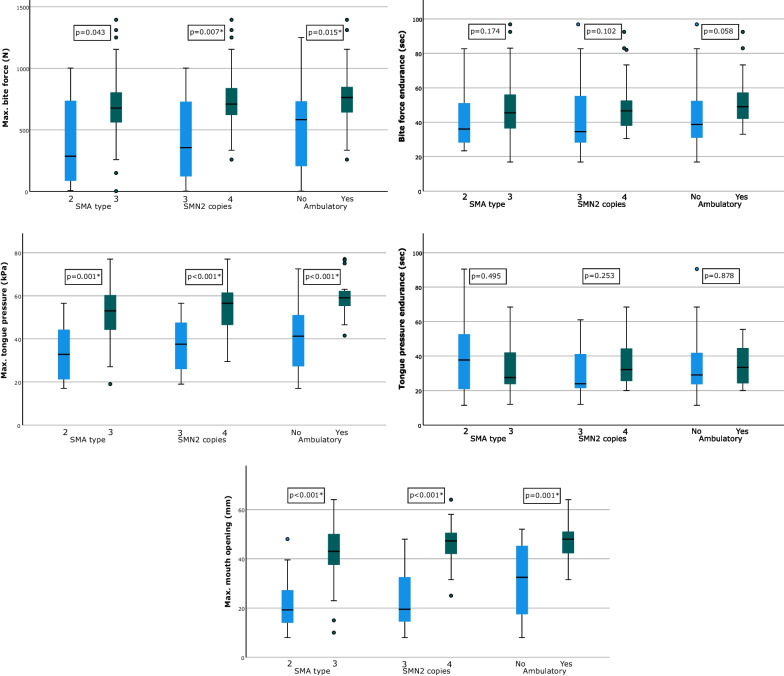
Differences in oral function across SMA type, *SMN2* copy number and ambulatory status. Box-plots including medians (thick lines), first and third quartiles (lower and upper limits of each box), minimum and maximum values (whiskers), as well as outliers (filled circles). Statistically significant distributional differences are indicated by an asterisk (*p* < 0.017; Bonferroni corrected). Top left: max. bite force, top right: bite force endurance, center left: max. tongue pressure, center right: tongue pressure endurance, bottom: max. mouth opening

Of the 43 individuals included, 25 were male and 18 were female. The mean age of the individuals at first testing was 39.7 ± 12.0 years (ranging between 20 and 65 years). Overall 60.5% of the individuals carried 4 *SMN2* copies, 34.9% carried 3 *SMN2* copies and one patient (2.3%) carried 2 *SMN2* copies. For one untreated patient information on the number of *SMN2* copies was missing (2.3%). According to clinical criteria, 12 individuals were diagnosed with SMA type 2 (one with 2 *SMN2* and 10 with 3 *SMN2* copies, one without information on the number of *SMN2* copies), and 31 with SMA type 3 (five with 3 *SMN*2, 26 with 4 *SMN*2 copies). The mean age of individuals for type 2 was 33.8 ± 8.3 years and for type 3 42.0 ± 12.5 years. The majority of the sample (*n* = 35) consisted of patients on nusinersen therapy who had received at least the first three doses according to routine clinical practice. Eight individuals were treatment-naive at the time of data collection, 28 individuals were non-ambulatory, and 15 were ambulatory at the time of examination (Table 1). In one case, a single motor score (HFMSE) could not be performed. Depending on the respective analysis, this missing information results in a sample size ranging between 41 and 43 (Fig. 1, Tables 2, 3 and 4).

**Table 1:** Sample characteristics

		SMA type 2		SMA type 3		Overall	
		Mean SD	*N* (%)	Mean SD	*N* (%)	Mean SD	*N* (%)
Age		33.8 ± 8.3	12 (27.9)	42 ± 12.5	31 (72.1)	39.7 ± 12	43 (100%)
Gender	Male		6 (50%)		19 (61.3%)		25 (58.1%)
	Female		6 (50%)		12 (38.7%)		18 (41.9%)
*SMN2* copies	2		1 (8.3%)		0 (0%)		1 (2.3%)
	3		10 (83.3%)		5 (16.1%)		15 (34.9%)
	4		0 (0%)		26 (83.9%)		26 (60.5%)
	Missing		1 (8.3%)		0 (0%)		1 (2.3%)
Ambulatory	Yes		0 (0%)		15 (48.4%)		15 (34.9%)
	No		12 (100%)		16 (51.6%)		28 (65.1%)
Nusinersen therapy	Yes		9 (75%)		26 (83.9%)		35 (81.4%)
	No		3 (25%)		5 (16.1%)		8 (18.6%)

**Table 2:** Median, minimum, and maximum values of oral function tests and clinical outcome scales

		Median	Minimum	Maximum
Oral function tests	Max. bite force (*N*)	665.2	2.7	1394.1
	Bite force endurance (sec)	44.5	17.0	96.9
	Max. tongue pressure (kPa)	48.0	17.0	77.0
	Tongue pressure endurance (sec)	29.0	11.5	90.5
	Max. mouth opening (mm)	42.0	8.0	64.0
Clinical outcome scales	BODS	2.0	2.0	6.0
	HFMSE	19.5	0.0	66.0
	RULM	23.0	0.0	56.0
	ALSFRS_R	34.0	14.0	46.0
	6MWT (m)	0.0	0.0	579.0

*BODS* Bogenhausener Dysphagia Score (higher scores indicate more severe dysphagia); *HFMSE* Hammersmith Functional Motor Scale; *RULM* Revised Upper Limb Module; *ALSFRS-R* Amyotrophic Lateral Sclerosis Functional Rating Scale Revised; *6MWT* 6-min-walk test

**Table 3:** Correlations among different parameters of oral function

		Max. bite force	Bite force endurance	Max. tongue pressure	Max.tongue pressure endurance
Bite force endurance	Spearman’s *ρ*	0.291			
	*p*-value	0.058			
	*N*	43			
Max. tongue pressure	Spearman’s *ρ*	0.439**	0.273		
	*p*-value	0.003	0.076		
	*N*	43	43		
Tongue pressure endurance	Spearman’s *ρ*	0.144	0.274	0.071	
	*p*-value	0.357	0.075	0.605	
	*N*	43	43	43	
Max. mouth opening	Spearman’s *ρ*	0.415**	0.295	0.558**	0.103
	*p*-value	0.006	0.055	< 0.001	0.511
	*N*	43	43	43	43

*ρ* = Correlation coefficient

**Correlation is stat. significant at the 0.01 level (two-tailed tests)

**Table 4:** Correlations between oral function and established clinical outcome scales

		BODS	HFMSE	RULM	ALSFRS-R	6MWT
Max. bite force	Spearman’s *ρ*	− 0.434*	0.384	0.372	0.388	0.409*
	*p*-value	0.004	0.012	0.014	0.010	0.006
	*N*	43	42	43	43	43
Bite force endurance	Spearman’s *ρ*	− 0.265	0.236	0.162	0.185	0.277
	*p*-value	0.085	0.132	0.299	0.236	0.072
	*N*	43	42	43	43	43
Max. tongue pressure	Spearman’s *ρ*	− 0.571*	.596*	0.668*	0.602*	0.580*
	*p*-value	< 0.001	< 0.001	< 0.001	< 0.001	< 0.001
	*N*	43	42	43	43	43
Tongue pressure endurance	Spearman’s *ρ*	− 0.141	0.030	0.030	0.149	0.046
	*p*-value	0.367	0.850	0.851	0.341	0.771
	*N*	43	42	43	43	43
Max. mouth opening	Spearman’s *ρ*	− 0.557*	0.703*	0.705*	0.661*	0.482*
	*p*-value	< 0.001	< 0.001	< 0.001	< 0.001	0.001
	*N*	43	42	43	43	43

*ρ* = Correlation coefficient

*Correlation is stat. significant at the 0.01 level (two-tailed tests; Bonferroni corrected)

BODS Bogenhausener Dysphagia Score (higher scores indicate more severe dysphagia); HFMSE Hammersmith Functional Motor Scale; RULM Revised Upper Limb Module; ALSFRS-R: Amyotrophic Lateral Sclerosis Functional Rating Scale Revised; 6MWT: 6-min-walk test

Table 2 gives a first impression of the measurements indicating the sample median values of the oral function tests and the clinical outcome scales. Minimum and maximum values show that both the oral function tests and the clinical outcome scales are subject to considerable variation in the sample of SMA patients.

Discriminant power was supported by distributional differences in the expected direction along SMA type, *SMN2* copy number and ambulatory status (Fig. 1). With the exception of tongue pressure endurance, all oral function measures tended toward higher values in patients with SMA type 3 relative to SMA type 2 (*n* = 43), in individuals with 4 relative to 3 *SMN2* copies (*n* = 41; one individual with 2 *SMN2* copies excluded for this analysis), and in ambulatory relative to non-ambulatory individuals (*n* = 43).

For maximum bite force, statistically significant distributional differences after correction for multiple comparison were observable between individuals with different *SMN2* copies (z = 2.653, *p* = 0.007) and different ambulatory status (z = 2.421, *p* = 0.015). Between individuals with SMA types 2 and 3, distributional differences in maximum bite force were statistically insignificant after Bonferroni correction (z = 2.031, *p* = 0.043). For maximum tongue pressure, statistically significant distributional differences after correction for multiple comparison were observable between individuals with different SMA types (z = 3.169, *p* = 0.001), different *SMN2* copies (z = 3.749, *p* < 0.001), and different ambulatory status (z = 4.078, *p* < 0.001).

For bite force endurance, all comparisons of distributional differences failed to reach statistical significance at a level of *p* < 0.017 Bonferroni corrected (all z ≤ 1.899). The same applied to all comparisons of distributional differences of tongue pressure endurance (all z ≤ 1.164).

For maximum mouth opening, statistically significant distributional differences after correction for multiple comparison were observable between individuals with different SMA type (z = 3.712, *p* < 0.001), different *SMN2* copies (z = 4.631, *p* < 0.001), and ambulatory status (z = 3.200, *p* = 0.001).

The correlation between maximum bite force and maximum tongue pressure was fair in size and statistically significant at the 0.05 level (*ρ* = 0.439, *p* = 0.003). Maximum mouth opening correlated fairly and statistically significantly with maximum bite force (*ρ* = 0.415, *p* = 0.006) and moderately with maximum tongue pressure (*ρ* = 0.558, *p* < 0.001). The two endurance-related measures showed little assessment agreement: Correlations between bite force endurance and tongue pressure endurance were fair, but statistically insignificant at the 0.05 level (*ρ* = 0.274, *p* = 0.075). The correlations between endurance measures and absolute maximum measures of muscle strength were poor to fair and statistically insignificant (*p* > 0.05; Table 3).

Maximum mouth opening correlated fairly with the 6MWT (*ρ* = 0.482), moderately with BODS, HFMSE, RULM and ALSFRS-R (all |*ρ*| ≥ 0.557). The correlations found between maximum mouth opening and all clinical outcome scales were statistically significant including the negative correlation with the Bogenhausener Dysphagia Score (BODS: higher scores indicate more severe dysphagia; all *p* < 0.01 Bonferroni corrected). The correlation between maximum tongue pressure and RULM was moderate and statistically significant (*ρ* = 0.668, *p* < 0.01 Bonferroni corrected). The correlations between maximum tongue pressure and BODS, HFMSE, ALSFRS-R, 6 MWT were fair and statistically significant after Bonferroni correction (all |*ρ*| ≥ 0.571, *p* < 0.01). Maximum bite force correlated fairly and statistically significantly at the 0.05 level with the HFMSE, RULM, ALSFRS-R, but statistically insignificantly after Bonferroni correction (all *ρ* ≥ 0.372, *p* ≥ 0.01). The correlations between maximum bite force and BODS as well as 6MWT were fair and still statistically significant at the 0.01 level after Bonferroni correction (both |*ρ*| ≥ 0.409,* p* ≤ 0.006)  . Correlations between tongue pressure endurance and clinical outcome scales were poor or almost non-existent and statistically insignificant (all |*ρ*| ≤ 0.149, *p* ≥ 0.341). Correlations between bite force endurance and clinical outcome scales were poor to fair (all |*ρ*| ≤ 0.277, *p* ≥ 0.072). All respective correlation coefficients (*ρ*) are shown in Table 4.

## Discussion

In this prospective, cross-sectional multicenter study, we for the first time addressed the evaluation of a set of oral function tests in adult SMA patients. New disease-modifying treatment options call for objective and sensitive methods to identify motor improvement in adult SMA patients. In recent years, the evaluation of bulbar function gained more attention and increasing interest in research [7, 16, 17, 26, 41]. Measuring small changes in adult SMA patients is complex and the combined use of several outcome measures is recommended—particularly so for specific subgroups. Oral function tests are suitable for complementing established motor scores in order to address their methodological limitations, but also to appropriately describe bulbar function in individuals at different functional levels and ages. The advantages of quantitative strength measures have been shown in ambulatory and non-ambulatory SMA patients [42–45]. Practically, the presented quantitative oral function tests have shown to be time-efficient given that they are bedside functional scores, and that muscle strength and endurance can be recorded with the same instruments.

In this study, we demonstrated that absolute maximum measures of oral function tests can discriminate between the various diagnostic types of SMA and between ambulatory and non-ambulatory individuals. They could differentiate between individuals with 3 or 4 *SMN2* copies, a time-constant parameter (i.e., irrespective of patients’ age and progression of the disease) that has been used in previous validation studies [46, 47]. The correlations between the absolute maximum measures and the clinical outcome scales were fair to moderate and therefore in expected ranges, notably because at best moderate correlations can be achieved when comparing survey methods that do not measure the same constructs [48]. The highest correlation coefficients were found for maximum mouth opening, confirming that maximum mouth opening is strongly correlated with SMA type, number of *SMN2* copies, maximum bite force, maximum tongue pressure and all clinical outcome scales. Restricted mouth opening is known to be associated with atrophy and fatty infiltration of the lateral pterygoid muscle [17] as well as (self reported) bulbar problems in individuals with SMA [17, 49]. Our findings support the idea that bulbar involvement is particularly well reflected in the measurement of maximal mouth opening. This may be because mouth opening is, compared to swallowing and chewing, mediated by a relatively limited number of muscles [17]. Similarly promising is the measurement of maximum tongue pressure, given the outlined correlations with established motor scores. From a clinical perspective, both methods are cost-effective and rather easy to handle for routine use.

In our data, there was little variation in the HFMSE values of weakest sitters (HFMSE < 5). These known problems of discrimination, not only of HFMSE but also of other motor scores [44, 50, 51], cannot yet be compensated by any valid outcome measure. This problem becomes particularly obvious during the survey of the 6MWT, where an evaluation is impossible for non-walkers. In contrast, oral function tests could be performed without any difficulties in this subgroup (e.g., maximum bite force ranging between 2.7 N and 1394.1 N in this study). The limited variability of the established motor scores at the lower end of the scales may reduce the statistical power of the data and could hence be the reason that not even higher correlations between the results of oral function tests and for example HFMSE, RULM or 6MWT could be achieved. The same holds true for the limited variability in BODS, where 84% of the individuals scored at 2. A more detailed analysis of ceiling and floor effects of oral function tests compared to established motor scores may provide further insights in this regard.

The relatively weak correlations observed between oral function endurance tests themselves and with established motor scores should be interpreted with some caution. Constructs tested by established motor scores are related but not identical to fatigability in bulbar function not least due to the time-lagged degeneration of bulbar function. Previous work assessing other neuromuscular diseases underlined that fatigability and weakness are distinct features of motor (dys)function [52, 53]. Endurance has been shown to be weakly associated with strength in some muscles [52]. Anatomical differences might explain the unexpected weak association between our endurance tests and the 6MWT, which sensitively detects fatigue-related changes in ambulatory SMA patients, but aims at different muscle groups [54]. Endurance measurements had been shown to be less reliable and less meaningful than absolute strength measurements [15, 39]. But in less impaired individuals (i.e., without oral dysfunction), they may be more sensitive to first constraints. Changes in bite force endurance or tongue pressure endurance could possibly contribute to a better discrimination in some subgroups, as prominent fatigability despite preserved muscle function has recently been detected in SMA patients [55]. The etiology of fatigability is complex. Fatigability and fatigue in SMA have been discussed as a secondary manifestation of impaired motor neurons and muscle loss, to be caused by neuromuscular transmission failure or by metabolic dysfunction [55–61]. Further attempts to determinate the reliability and to establish endurance tests are crucial steps to better understand fatigability (of bulbar function) in SMA and should be pursued.

Anatomical conditions of SMA patients and methodological challenges complicated the measurement of bite force and tongue pressure and especially endurance measurements. Similar to other neuromuscular diseases, the altered craniofacial development in SMA patients leads to a vertical growth pattern with an anterior open bite and a narrow and deep palate [15, 62]. In the case of an anterior open bite or other malocclusions, the measurement of maximum mouth opening may have been compromised and could have led to overly high values in our examinations. Due to the high palate, SMA patients may not have been able to apply full tongue pressure to the air-filled bulb or, more important, hold the tongue position for a longer time period. The challenging positioning of the air-filled bulb itself may also have added inaccuracies, as the bulb tended to slide on the tongue surface [63]. Just as bite force decreases with greater interocclusal distance [64], tongue pressure and endurance may have been influenced by the interincisal separation which is caused by the thin flexible connector tube of the IOPI device [65, 66]. To reduce interincisal separation, a thin intraoral sensor was chosen for maximum bite force measurements. While the adjusted soft sensor surface improved the area under load and prevented subconscious inhibition due to periodontal or temporomandibular joint sensibility [35, 67], it resulted in a slightly increased interocclusal distance. This distance unavoidably varied between patients which may have led to minor inaccuracies. For endurance measurements, we chose a target value of 60% of the previously determined force. Although this procedure was in line with previous approaches [15], it can be questioned for two reasons. First, methodologically inaccurate maximum values can translate into biased endurance measurements. Second, if patients had been asked to generate an absolute predefined force level, interindividual differences would potentially have been more pronounced.

The heterogeneity of patients, sometimes seen as a limitation reducing the analytic power of the data, allowed us to assess the reliability and applicability of oral function tests across a wide range of individuals differing in SMA type and *SMN2* copies. These wide-ranging data on oral function in SMA have the potential to reveal additional insights into bulbar involvement in SMA through further subgroup analyses.

As only cross-sectional data were analyzed in this study, there was no possibility to examine the relative sensitivity of oral function tests to changes over time. Further research is necessary to confirm oral function tests as a standardized outcome measure in the clinical evaluation of SMA patients.

## Conclusions

This study provides support for continued investigation on oral function using maximum bite force and maximum tongue pressure measurements, complemented by endurance tests and the evaluation of maximum mouth opening. The measurement of maximum mouth opening and tongue pressure, and to a lesser degree also maximum bite force have shown to be appropriate instruments both as clinical measures as well as outcome measures for clinical trials in individuals with SMA. In particular, they are able to meaningfully supplement existing motor scores in specific questions about bulbar function or in certain subgroups as severely affected, non-ambulatory individuals.

## Data Availability

The datasets used and/or analyzed for the current study are available from the corresponding author on reasonable request.
